# Right aural myiasis in a hypertensive patient: a rare and disturbing ear, nose, and throat emergency (case report)

**DOI:** 10.11604/pamj.2026.53.60.49829

**Published:** 2026-02-05

**Authors:** Rupali Warghane, Ruchira Ankar, Arti Raut, Samrudhi Gujar, Swapna More

**Affiliations:** 1 Shrimati Radhikabai Meghe Memorial College of Nursing, Datta Meghe Institute of Higher Education and Research, Sawangi (Meghe), Wardha, Maharashtra, India

**Keywords:** ENT emergency, ear maggots, aural myiasis, larvae infestation, case report

## Abstract

Aural myiasis is an uncommon parasitic infestation involving the external auditory canal by fly larvae, typically seen in tropical climates, especially among individuals with poor hygiene or immunocompromised status. Though rare, it can lead to serious complications, including tympanic membrane perforation or intracranial invasion. We describe a 55-year-old man with hypertension who had been experiencing pain and bleeding in his right ear for four days. Live maggots were found in the right external auditory canal during otoscopic examination. It was impossible to see the tympanic membrane. Systemic and topical antibiotics, supportive therapy, and aural toileting were all effective treatments for the patient. To avoid major complications, aural myiasis must be diagnosed early and treated quickly. In this instance, there was no deeper structure infection or involvement of the facial nerve, and the recovery went smoothly. The patient was receiving antihypertensive medication and had a history of angioplasty. Even though it is uncommon, patients who present with sudden-onset ear pain, bleeding, and a crawling sensation should be evaluated for aural myiasis, particularly in areas where it is endemic. For management, early detection and comprehensive debridement are essential.

## Introduction

People with poor hygiene, low socioeconomic status, or weakened immune systems, including the elderly and those with long-term ear disorders, are usually affected. The larvae cause symptoms like severe ear pain, itching, hearing loss, and foul-smelling discharge because they feed on the tissue and secretions inside the ear canal [1]. Serious consequences like tympanic membrane perforation, secondary bacterial infections, and, in extreme situations, intracranial invasion that results in potentially fatal illnesses like meningitis or brain abscess can arise from the infestation if treatment is not received [2]. During otoscopic examination, the larvae are typically directly visualized to make the diagnosis. The larvae are then carefully mechanically removed, immobilized with topical agents, and treated with antibiotics to control secondary infections [3]. Preventive measures include minimizing fly exposure, especially in endemic areas, managing ear infections quickly, and practicing good ear hygiene [4].

## Patient and observation

**Patient information:** a 55-year-old male patient, known to have hypertension, is currently on medications including Metoprolol and Rosuvastatin. He has a significant past medical history of undergoing angioplasty 15 years ago. The patient does not have a history of diabetes mellitus, tuberculosis, or bronchial asthma.

**Chief complaints (duration):** the patient presented with right ear pain and bleeding for the past four days. The pain started suddenly five days ago, described as continuous, sharp, and throbbing, accompanied by itching and a crawling sensation in the right ear. After three days, the patient noticed moderate fresh bright red bleeding from the same ear. There were no factors identified that worsened or relieved the symptoms. The patient denied any history of trauma, ear instrumentation, or recent upper respiratory tract infection. Additionally, there were no associated Ear, Nose, and Throat (ENT) symptoms such as giddiness, tinnitus, nasal obstruction, nasal discharge, nasal bleeding, throat pain, difficulty swallowing, voice changes, or facial weakness.

### Clinical findings

**General examination:** the patient was found to be afebrile with a pulse rate of 80 beats per minute and a blood pressure of 120/80 mmHg. The patient´s height was 165 cm, weight 55 kg, resulting in a BMI of 20.2.

**Figure 1:**
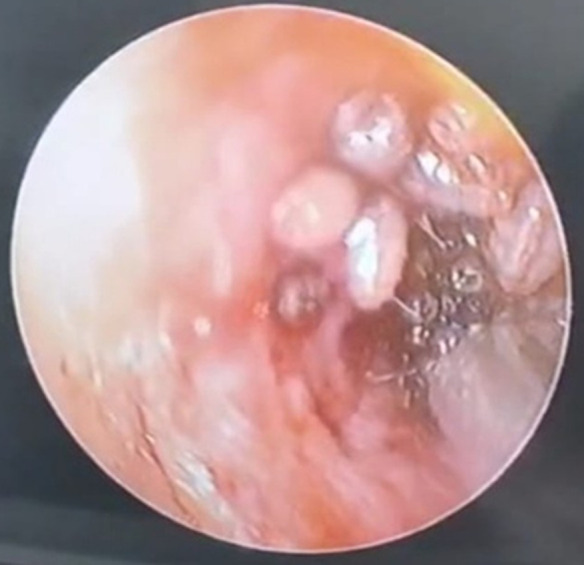
maggots viewed through the otoscope

**Ear, nose, and throat examination:** revealed bleeding from the right external auditory canal, where maggots were visualized, and the tympanic membrane was not visible. The left ear showed a normal external auditory canal and an intact tympanic membrane. There were no clinical signs of facial nerve palsy. Nasal examination showed a deviated nasal septum to the right side along with bilateral inferior turbinate hypertrophy. The oral cavity and throat examination were unremarkable with normal findings (Figure 1).

### Diagnostic assessment

**Laboratory tests:** initial laboratory investigations revealed mild leukocytosis, which showed an improving trend over time. Platelet count was initially low at 55,000 but later normalized. Peripheral smear demonstrated normocytic, normochromic red blood cells with no evidence of parasitic infection. Urinalysis showed trace amounts of albumin, with the presence of many budding yeast cells and non-squamous epithelial cells, suggesting a possible fungal urinary tract infection. Liver function tests (LFT), kidney function tests (KFT), and random blood sugar (RBS) were all within normal limits. The coagulation profile was normal with an INR of 1.01. Dengue serology was negative. Imaging studies, including a chest X-ray and electrocardiogram (ECG), were within normal limits.

**Therapeutic intervention:** the inpatient treatment plan included intravenous antibiotics with Inj. Augmentin 1.2 g administered every 12 hours for 7 days and Inj. Gentamycin 80 mg twice daily for 3 days. Oral medications comprised Tab Rabemac 20 mg twice daily for 7 days, Tab Zerodol SP twice daily for 7 days, Tab Chymoral Forte three times daily for 3 days, Tab Elriz XL once at bedtime for 7 days, and Tab Nicardia 10 mg twice daily, continued for hypertension. The ear drops prescribed were Waxonil, two drops three times daily for 7 days, Botroclot, two drops three times daily for 7 days, and Otobiotic, two drops three times daily for 2 days. Additionally, syrup Duphalac 20 ml was given at bedtime for 5 days to aid bowel movements. Aural toileting involved mechanical removal of maggots under otoscopic guidance, along with local irrigation using antiseptic and antibiotic solutions to ensure thorough cleaning and infection control.

**Follow-up and outcomes:** the patient responded well to the treatment, with no recurrence of bleeding or pain. Repeat otoscopic examination revealed a clean external auditory canal with no live maggots present. However, the tympanic membrane was still not visualized due to persistent oedema. There were no signs of neurological involvement or systemic infection throughout the course of treatment.

**Patient perspective:** the patient reported total symptom relief following removal and treatment, despite expressing considerable anxiety upon finding maggots in the ear. He was compliant with treatment and follow-up and stressed the value of receiving medical attention as soon as possible.

**Informed consent:** the patient gave written informed consent for this case report and its clinical details to be published.

## Discussion

A rare but potentially dangerous condition called aural myiasis is brought on by fly larvae infesting the outer ear canal. Because warm, humid weather encourages fly reproduction, it is primarily found in tropical and subtropical regions. Affected individuals are more likely to have poor hygiene, low incomes, or weakened immune systems, such as the elderly or those with long-term ear issues [5].

In this instance, a 55-year-old man with hypertension arrived at the hospital with the usual signs of aural myiasis, which included pain in his right ear, bleeding, and a feeling of maggots crawling inside his ears. Other studies have reported similar symptoms, and for instance, reported patients who experienced ear pain, foul-smelling discharge, and occasionally eardrum damage. Chronic middle ear infection can cause persistent foul-smelling ear discharge and hearing loss. This is due to a perforation of the eardrum and recurrent infection of the middle ear. The ear perforation is usually the result of previous trauma or infection [6]. Our patient's symptoms were like common early indicators, such as itching, ear discharge, and a sensation of movement in the ear. Blood tests for the patient revealed a high white blood cell count and somewhat low platelets, both of which improved with treatment.

The body's response to the parasite and any subsequent infection was probably reflected in these outcomes. Additionally, it was discovered that patients with more severe cases frequently had higher levels of inflammation, highlighting the importance of prompt treatment, as parasites disrupt CNS function and systemic inflammation as a critical link between peripheral infections and neuroinflammatory conditions, advancing understanding of parasite-associated neurological complications [7]. The diagnosis was confirmed when maggots were found during an ear examination, which is still the most reliable way to identify the condition. Although imaging was not used in this case, Kumar *et al*. state that high-resolution CT scans can be useful in complex cases to check for deeper ear involvement or bone damage [8].

The larvae were manually removed as part of the treatment, and oral and topical antibiotics were administered. Common bacteria like *Pseudomonas aeruginosa* and *Staphylococcus aureus* were combated with broad-spectrum antibiotics like Augmentin and Gentamycin. Topical medications like Waxonil and Botroclot also assisted in halting bleeding and controlling infection. Local swelling following the infestation occasionally made it challenging to see the eardrum clearly. Fortunately, there were no severe side effects, which are uncommon but possible and could include nerve damage or infection spreading to other areas of the body. The significance of keeping an eye out for indications of these complications, particularly in patients with compromised immune systems or untreated infections. Ultimately, increasing healthcare professionals' knowledge of the early signs and proper treatment of aural myiasis can improve patient outcomes. Regular otoscopic examinations for people who are at high risk, along with public health measures that stop the spread of this rare but serious disease, are important for lowering the number of cases [9]. Reducing fly exposure in areas where aural myiasis is prevalent, treating ear infections promptly, and practicing good personal hygiene are all important ways to prevent the condition. Cases can be reduced by public health initiatives that increase awareness among vulnerable populations [10].

## Conclusion

In endemic regions, patients with acute onset of otalgia, otorrhagia, and crawling sensation should be evaluated for aural myiasis, despite its rarity. Complications can be avoided with a thorough examination and prompt intervention. Multidisciplinary cooperation and proper ENT care in this instance guaranteed a successful resolution.
